# Novel Alanyl-tRNA Synthetase 2 Pathogenic Variants in Leukodystrophies

**DOI:** 10.3389/fneur.2019.01321

**Published:** 2019-12-17

**Authors:** Xingao Wang, Qun Wang, Hefei Tang, Bin Chen, Xiang Dong, Songtao Niu, Shaowu Li, Yuzhi Shi, Wei Shan, Zaiqiang Zhang

**Affiliations:** ^1^Department of Neurology, Beijing Tiantan Hospital, Capital Medical University, Beijing, China; ^2^China National Clinical Research Center for Neurological Diseases, Beijing, China; ^3^Department of Neurology, Epilepsy Center, Beijing Tiantan Hospital, Capital Medical University, Beijing, China; ^4^Beijing Institute for Brain Disorders, Beijing, China; ^5^Department of Neurology, The First Affiliated Hospital of Dalian Medical University, Dalian Medical University, Dalian, China; ^6^Beijing Institute of Neurosurgery, Beijing, China

**Keywords:** leukodystrophies, *AARS2*, pathogenic variants, white matter, mutation

## Abstract

The white matter disease spectrum is associated with many genetic diseases, including *AARS2, CADASIL, ALD*, and others. In this study, to determine the novel *alanyl-tRNA synthetase 2* mutation implicated in white matter disease, several families with an autosomal recessive inheritance pattern of white matter disease were analyzed by whole-exome sequencing. Variants were prioritized according to their rarity and pathogenic variants in genes already known to be associated with leukodystrophies and were confirmed by Sanger sequencing using standard protocols. We identified 5 rare variants (c.452T>C chr6:44279256 p.M151T, c.1871G>A chr6:44272054 p.W624X, c.802A>G chr6:44278128 p.M268V, c.1703-1704del chr6:-44272430-44272431 p.Q568fs, and c.179C>A chr6-44280882 p.P60H) with varying expression in 4 independent Chinese families with leukodystrophy. These single nucleotide variants (SNVs), or deletion mutations, each induced a frameshift, causing a missense mutation in *alanyl-tRNA synthetase 2*. These findings suggested that all mutations might contribute to the development of leukodystrophy in the examined family members. Combined with previous findings, our data confirmed that the novel mutations are located in leukodystrophy-related risk genes. We also summarized all the *alanyl-tRNA synthetase 2* mutations related to the onset of leukodystrophies in adults.

## Introduction

Leukodystrophy is a genetic and progressive multifocal neurological syndrome of the white matter in the central nervous system (1), first described in 1958 by Astrom as a human demyelinating disease (2). In the 1990s, multiple genetic factors were discovered as etiological causes of leukodystrophies. Currently, more than 100 genetic factors have been identified and reported to be associated with leukodystrophies (3–9). Diagnosing leukodystrophy is challenging and requires clinical, radiographic, and genetic evidence. According to previous studies, only half of patients are clearly diagnosed with leukodystrophies as the cause of disease (10).

Leukodystrophies are caused, in part, by mitochondrial respiratory chain dysfunction and mitochondrial energy metabolism disorders (11, 12). Since the mitochondrial respiratory chain oxidative-phosphorylation (OXPHOS) activity is under the dual control of mitochondrial DNA (mtDNA) and nuclear DNA (12), mitochondria-induced white matter disease can be caused by mtDNA mutations (such as mitochondrial myopathy, encephalopathy, lactic acidosis, stroke-like episodes, MELAS) or by nuclear DNA mutations (such as mitochondrial neurogastrointestinal encephalopathy, MNGIE). A large amount of new protein is required for ATP production through mitochondrial electron transport and OXPHOS activity, including promoter, extension, and termination factors for mitochondrial translation, tRNA-modifying enzymes, and mitochondrial aminoacyl-tRNA synthases (8).

Aminoacyl-tRNA synthase is responsible for recognizing specific tRNAs linked with the corresponding amino acids, initiating the synthesis of peptide chains (13). Since the discovery of aspartic tRNA synthase deficiency (*DARS2*) in leukodystrophies with brain stem and spinal cord involvement and lactate elevation (LBSL) in 2007 (9), there have been 14 reported pathogenic mutations that encode the mitochondrial aminoacyl-tRNA synthase gene in neurological diseases, such as mt-AlaRS (*AARS2*), mt-AsnRS (*NARS2*), mt-AspRS (*DARS2*), mt-ArgRS (*RARS2*), mt-CysRS (*CARS2*), mt-GluRS (*EARS2*), mt-HisRS (*HARS2*), mt-IleRS (*IARS2*), mt-LeuRS (*LARS2*), mt-MetRS (*MARS2*), mt-PheRS (*FARS2*), mt-ProRS (*PARS2*), mt-ThrRS (*TARS2*), and mt-ValRS (*VARS2*) (14). These mutations can induce multiple neurological disorders, such as epilepsy, autosomal recessive spastic ataxia with leukodystrophy (ARSAL), distal hereditary motor neuropathy (dHMN), hereditary motor neuropathy (HMN), LBSL, pontocerebellar hypoplasia (PCH), and leukodystrophy with thalamic and brainstem involvement and hyperlactatemia (14). However, the *AARS2* mutation induces two different mutation-dependent diseases: ovarioleukodystropy (14, 15) and lethal mitochondrial cardiomyopathy with lactacidosis (16). Alanyl-transfer RNA synthetase 2 mutation-related leukodystrophy (*AARS2*-L) is an autosomal recessive neurodegenerative disorder related to *AARS2* gene mutation (15). In 2014, Dallabona et al. reported that *AARS2* mutations could lead to late-onset progressive white matter malnutrition, leading to premature ovarian failure in female patients (17). In subsequent studies, researchers from Japan and South Korea reported white matter disease caused by different *AARS2* mutations in Asian patients (18, 19). At present, a total of 15 sporadic *AARS2* mutation-related leukodystrophy patients (including eight females and seven males) have been reported around the world.

The clinical manifestations of progressive white matter disease were determined as novel *AARS2* compound heterozygous mutations or homozygous mutations by whole-exome sequencing, and we then summarized and analyzed all the mutations in *AARS2* related to adult-onset leukodystrophies.

## Materials and Methods

### Study Population

**Figure 1:**
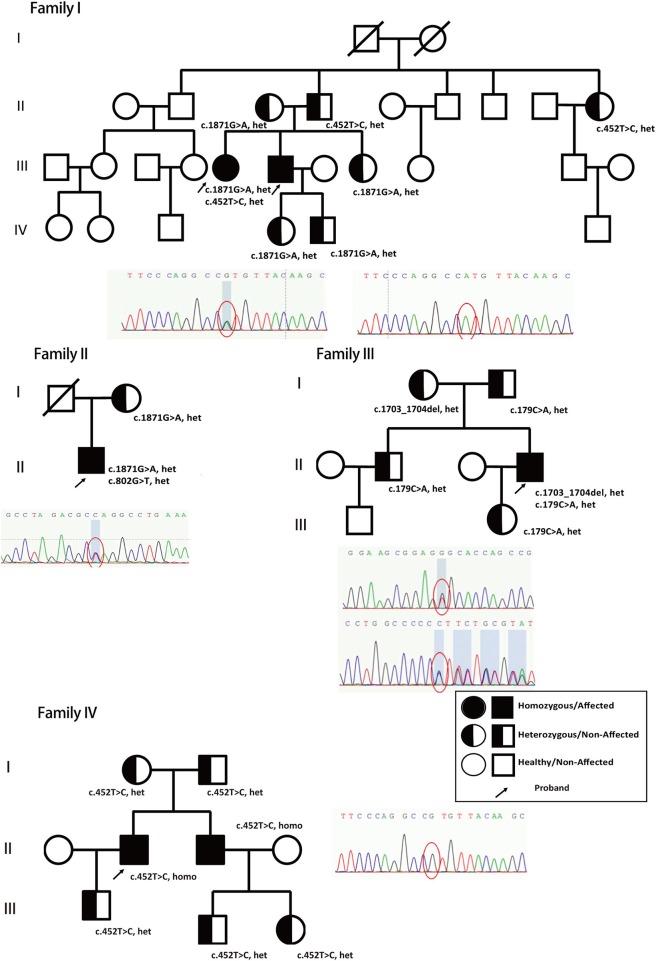
Genetic models and family trees. Patients' different novel nonsense heterozygous mutations (c.1871G>A, het, c.452T>C, het, from family I; c.1871G>A, het, c.802G>T, het, from family II; c.1703_1704del, het, c.179C>A, het from family III; and c.452T>C, homo, from family IV, marked with arrow) inherited from their parents and verified in other family members. The full shaded circle/square indicated affected female/male patients (Homozygous), half-shaded circle/square indicated non-affected female/male (heterozygous), non-shaded circle/square indicated healthy female/male. The arrow indicated the proband in the family who has been reported and confirmed clinically in this study.

In this study, we report another 5 *AARS*-L patients (four males and one female) from four different families in China. Five patients (P1, P2, P3, P4, and P5) gathered from four different families in China were included in this study (family maps are shown in Figure 1). P2, P3, and P5 were hospitalized patients, and the informed consent forms were signed by their family members. P4 was hospitalized in another hospital for detailed examination and provided consent for access to complete data. This study was approved by the Ethics Committee of the Beijing Tiantan Hospital and fulfilled the Helsinki Declaration.

P1 and P2 are from the same family, while P3, P4, and P5 are from different families. Acquired causes of white matter disease, such as infectious diseases, poisoning, and metabolism-related diseases, were systematically excluded in P2, P3, P4, and P5. Laboratory tests showed no immunological abnormalities, and imaging investigations did not indicate tumors.

### Muscle Biopsy

Muscle biopsy was performed on patients P2, P3, and P5 according to standard protocols and histopathological procedures. The muscle specimens collected from P3 were studied by transmission electron microscopy at the same time. However, due to personal reasons, P1 and P4 refused to undergo muscle biopsy, and P1, P2, P4, and P5 refused to undergo electron microscopy.

### Mitochondrial Histochemical Analysis and Enzyme Assay

Partial muscle biopsy specimens from P2 and P3 were homogenized, and the mitochondrial activities of succinate dehydrogenase (SDH), NASH-FeCN reductase, succinate ubiquinone reductase (SQR), succinate-cytochrome c reductase, cytochrome c oxidase, complex V ATP synthase, and citrate synthase (20, 21) were assayed as described previously by spectrophotometry and compared with non-mitochondrial muscle specimens detected at the same time. All assays were performed at 25°C using 50 mM potassium phosphate (pH 7.5) as a reaction buffer.

### Whole-Exome Sequencing (WES) and Sanger Sequencing

Genomic DNA extraction from collected tissues was carried out using the ChargeSwitch® gDNA Mini Tissue Kit (Life Technologies, Carlsbad, CA). The extracted DNA was used for construction of a library using a SOLiD Fragment Library Construction Kit (Life Technologies). The library DNA was subjected to whole-exome enrichment using a Sureselect Human All Exon Kit (Agilent Technologies, Inc., Santa Clara, CA). The enriched library DNA was then sequenced using the SOLiD System, a deep sequencer employing the massively parallel sequencing method, and analyzed using Bioscope software (Life Technologies). After excluding mutations such as *CSF1R* and *EIF2B*, they were finally identified as *AARS2* compound heterozygous mutations. Family members' gene mutations were verified by Sanger sequencing.

For validation of the WES data, DNA was amplified by polymerase chain reaction (PCR). The amplified products were treated with ExoSAP-IT (GE Healthcare, Chalfont St Giles, Buckinghamshire, UK) and sequenced using Bigdye Terminator and a 3130xl Genetic Analyzer (Life Technologies) for examination of mutations in *AARS2*.

## Results

### Clinical Manifestations and Laboratory Findings

Patients P1 and P2 from family 1 both have healthy parents. P1 is a 35-year-old female, the sister of the proband P2. She developed right hand tremors at the age of 22. Three years later, she developed spasticity, dystonia, dysarthria, and cognitive deterioration. At the age of 27, she had a reduction in menstruation frequency, with a menstrual cycle period once every 2–3 months, and later developed amenorrhea. Even though the clinical phenotype was classic, the patient refused to undergo further CT, MRI, and biopsy examinations.

Patient P2 is a 29-year-old male. His exercise capacity and intelligence were normal until 12 years of age. He started to experience cognitive deterioration in junior high school. At the age of 18, he developed an involuntary right hand tremor. At the age of 20, he was found to exhibit slow walking and difficulty running. At the age of 23, his speech became unclear. At the age of 28, he developed stiffness and weakness in the left lower limb. On examination, he had bilateral slow eyeball scan, nystagmus, and mild abducens nerve palsy, without double vision. Saccades were slowed. The patient also had dysarthria, and his bilateral pharyngeal reflex was absent. He walked with a paralyzed gait and was unable to walk straight. The muscular tension of all limbs was high and was relatively higher on the left side than on the right side. The bilateral Babinski sign was present. The patient had postural and intention tremors of the right hand. Bilateral mild pes cavus could be observed. The Montreal Cognitive Assessment (MoCA) score was 20/30. No cardiac abnormalities were observed in the physical examination. Electrocardiograms and echocardiograms were normal. Blood lactic acid was slightly high (3.3 mmol/l), and no abnormalities were reported on electroencephalogram (EEG) and electromyography (EMG).

Patient P3 is from family 2, and he is 26 years old. His father was infected with hepatitis B and eventually died of cirrhosis and liver cancer at the age of 50. His mother is 54 years old and healthy. The patient was normal at birth and experienced dyskinesia at the age of 3 months (100 days). His parents found that it was difficult for him to raise his head at 5 months. He was able to sit at 10 months, stand at 1.5 years, and walk at 3 years of age. His walking was slow and unstable. At the age of 4, the patient was able to walk independently but was unable to run. After the age of 12, his walking ability gradually improved, and he could drive when he was 23. At the age of 25, the patient developed numbness in his right upper and lower limbs and difficulty writing. No abnormalities were found on head CT at the corresponding time. Dysphagia and dysdipsia appeared 2 months later. After 4 months, the patient developed apathism and personality changes as well as speech ambiguity at the same time. On examination, he had bilateral slow eyeball scan, mild bilateral abducens nerve palsy, and vertical nystagmus. He also had dysarthria and weakened pharyngeal reflexes. Muscular tension was high in all the limbs and was more obvious in the bilateral lower limbs. The minuting fingers on both hands remained weak. The bilateral Babinski sign was present. Bilateral cerebellar ataxia was found, and a slight tremor in both hands and an unsteady gait were present. Bilateral mild pes cavus could be observed. The MoCA score was 19/30. No cardiac abnormalities were found. The blood lactate level was 3.4 mmol/l, the EMG showed myogenic changes in the bilateral deltoid muscles, and the EEG showed slow waves in the bilateral occipital areas.

Patient P4 is from family 3. The patient is a 31-year-old male. His parents are both healthy. He began to have memory impairment at the age of 29. At the same time, his right lower limb gradually became weak and stiff, which made him unable to run. Eighteen months later, he developed unclear speech, dysdipsia, urinary urgency, and incontinence. The patient also experienced personality changes, insomnia and dreaming for the past 2 years. On examination, he had a slow response. Bilateral mild abducens paralysis and visible horizontal nystagmus were observed. He also had dysarthria and weakened pharyngeal reflexes. Muscular tension was high in both lower limbs and was more obvious on the right side. The bilateral Babinski sign was present. The patient was unable to walk straight. The Romberg sign was positive, and the MoCA score was 24/30. EEG and ECG showed no cardiac abnormalities.

Patient P5 is from family 4. He is a 38-year-old male. His mother had a history of hypertension. His father has diabetes. The patient had dysthymia symptoms, including reduced discourse, slow response, less interpersonal communication with others, unwillingness to participate in social activities, and unwillingness to go outside. There were also personality changes since the onset of symptoms. On physical examination, he had a slow response. Bilateral mild abducens nerve palsy and visible horizontal nystagmus were observed. The patient also had dysarthria and weakened pharyngeal reflexes.

Muscular tension was high in both lower limbs and was more obvious on the right side. The bilateral Babinski sign was present. The patient was unable to walk straight. The Romberg sign was positive, and the MoCA test score was 8/30 (mainly impairment in visual space, execution ability, memory, attention, orientation, and abstract thinking). The MMSE test score was 11/30 (mild cognitive impairment). The Hamilton Depression Rating Scale test excluded depression and related disorders, and the Hamilton Anxiety Rating Scale test excluded anxiety and related disorders. Electrocardiogram and echocardiogram showed no cardiac abnormalities. The EMG test was normal. According to the genetic prediction, the brother of patient P5 should be homozygous; however, he is healthy and without any of the clinical symptoms exhibited by P5.

### WES and Sanger Sequencing

**Figure 2:**
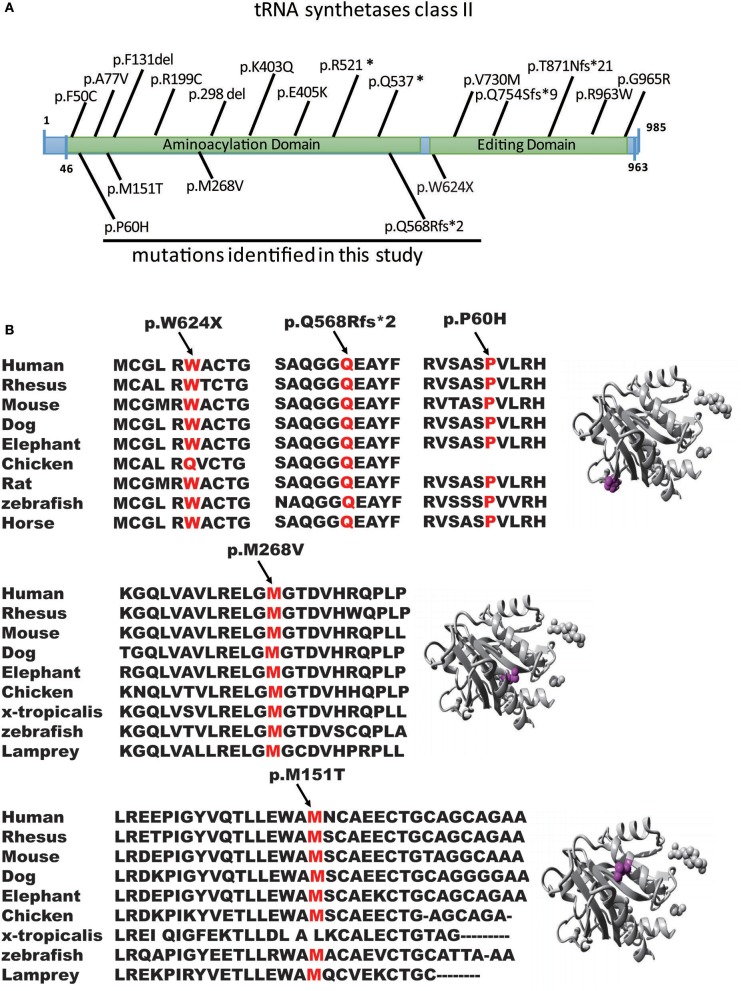
Genomic structure of AARS2. **(A)** Genomic structure of AARS2 with exons coding for the aminoacylation and editing domains. The arrows indicate the position of mutations identified in this study (bottom) or previously reported (top). **(B)** Conservation level of the positively charged residues in AARS2-L, which are predicted to be involved in tRNA-Ala recognition in human AARS. Domains are color-coded, and mutations are marked in orange.

The patients' genomic DNA was extracted from their blood samples, followed by WES. The sequencing data confirmed that the *ABCD1, CSF1R, EIF2B*, and *NOTCH3* genes were mutation-free. Adrenoleukodystrophy (ALD), adult-onset leukodystrophy with axonal spheroids and pigmented glia (ALSP), vanishing white matter disease (VWM), and cerebral autosomal dominant arteriopathy with subcortical infarcts and leukodystrophy (CADASIL) were all preemptively excluded to determine *AARS2* complex heterozygous mutations. Patient P2 had a c.1871G>A, p.W624X and c.452T>C, p.M151T compound heterozygous mutation, and the family reported that patient P1 carried the same compound heterozygous mutation. The patient's father carried only the c.452T>C mutation, while his mother had only the c.1871G>A mutation. The parents of patient P2 were heterozygous, and their sisters, aunts, and children all had heterozygous mutations (Figure 1, Family I, Figure 2).

Patient P3 had a c.1871G>A, p.W624X, and c.802A>G, p.M268V compound heterozygous mutation. The mother was confirmed to carry the c.1871G>A, p.W624X heterozygous mutation, and the deceased father may also have carried c.802A>G, p.M268V heterozygous mutations (Figure 1, Family II, Figure 2).

Patient P4 had a c.1703-1704del, p.Q568fs, and c.179C>A, p.P60H compound heterozygous mutation. Family validation showed that her parents, older brothers and daughters all exhibited heterozygous mutations (Figure 1, Family III, Figure 2).

Patient P5 had a c452T>C, p.M151T homozygous mutation. Family validation confirmed that his parents also had heterozygous mutations, and so did his brother, his son and his nephews, with all of them showing heterozygous mutations (Figure 1, Family IV).

### Muscle Biopsy

Patients P2, P3, and P5 underwent muscle biopsy examination. P2's sample was studied using transmission electron microscopy.

**Figure 3:**
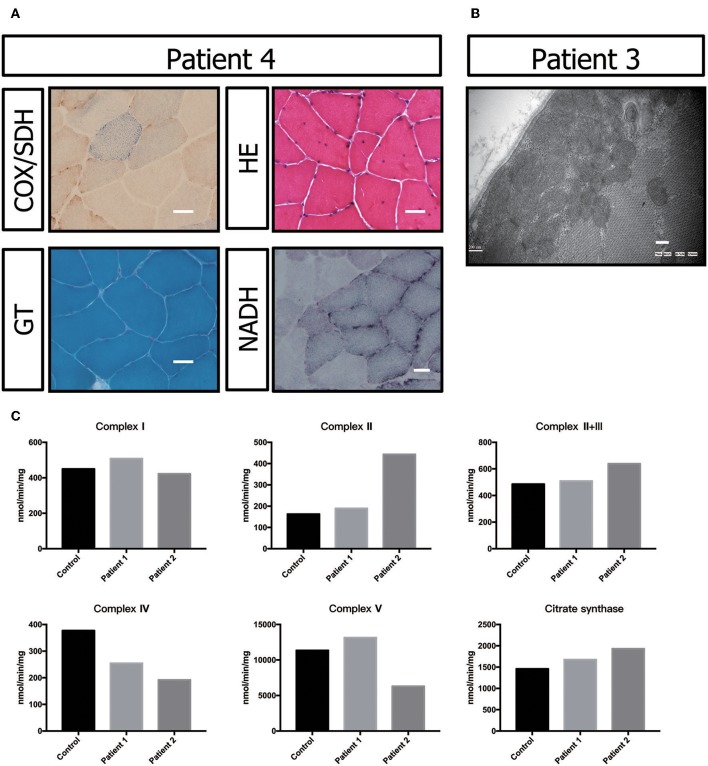
Biochemical and genetic features. **(A)** Morphological analysis of muscle biopsy specimens from the healthy control and patient P3: Gomori trichrome (GT) staining and cytochrome c oxidase (COX)/succinate dehydrogenase (SDH) histoenzymatic double staining (COX/SDH). **(B)** Several mitochondria were observed by transmission electron microscopy to accumulate under the sarcolemma in patient P5. **(C)** Biochemical activities of mitochondrial respiratory chain complexes in muscle homogenates from patients P3 and P4. All enzymatic activities are normalized for citrate synthase activity and are indicated as percentages relative to the mean control value.

Muscle biopsy showed no ragged-red fiber (RRF) with modified Gomori trichrome (MGT) staining. Negative fibers were shown with cytochrome oxidase (COX)/succinic dehydrogenase (SDH) staining. Hematoxylin-eosin (HE) staining primarily showed a nucleus shift, muscle fiber grouping phenomenon and small angular atrophic fibers (Figure 3A). All these results indicated mild neurogenic damage in patient P5. In patients P2 and P3, the muscular pathological changes under light microscopy were similar to those described in previous reports (data not shown) (17). However, transmission electron microscopy showed a large number of accumulated mitochondria under the sarcolemma (Figure 3B).

The electron transport chain enzyme activity in the muscle specimens from P2 and P3 showed reduced muscle mitochondrial complex IV activity compared to that in the control group. The decrease in mitochondrial complex IV activity in patient P3 was more pronounced (Figure 3C, Table 1).

**Table 1:** Electron transport chain enzyme activity in patients' muscle specimens.

**ETC complexes**	**Activity (nmol/min/mg)**		
	**Controls**	**P2**	**P3**
Complex I NASH-FeCN reductase	449.6	508.4	422.7
Complex II Succinate dehydrogenase	162.8	189.6	443.7
Complex II + III Succinate-cytochrome c reductase	485.4	509.8	638.8
Complex IV Cytochrome c oxidase	377.0	254.4	192.2
Complex V ATP synthase	11357.6	13154.7	6310.4
Citrate synthase	1459.0	1678.4	1931.8

### Neuroimaging

Patients P2, P3, P4, and P5 underwent a head CT scan, which did not show signs of calcifications.

The brain MRI of P2 showed abnormal signals on T2-weighted imaging in the ventricular white matter, the posterior horn of the lateral ventricle, the corpus callosum, the posterior branch of the internal capsule and the cerebral peduncle. These signals showed high, uneven intensity on fluid-attenuated inversion recovery (FLAIR) imaging. On T1-weighted imaging, the corpus callosum was obviously shrunken and thinned and showed sandwich-like changes. The corpus callosum splenium showed band-like abnormalities. Diffusion-weighted imaging (DWI) showed paraventricular and intracapsular posterior branch spot-like diffusion restriction. Magnetic resonance spectroscopy (MRS) showed an inverted double lactate peak. MR angiography (MRA) showed normal cerebral blood vessels. Susceptibility weighted imaging (SWI) demonstrated no evidence of microbleeding, and no contrast enhancement was found (Figure 3). P2's father and child showed normal head MRI findings (data not shown).

**Figure 4:**
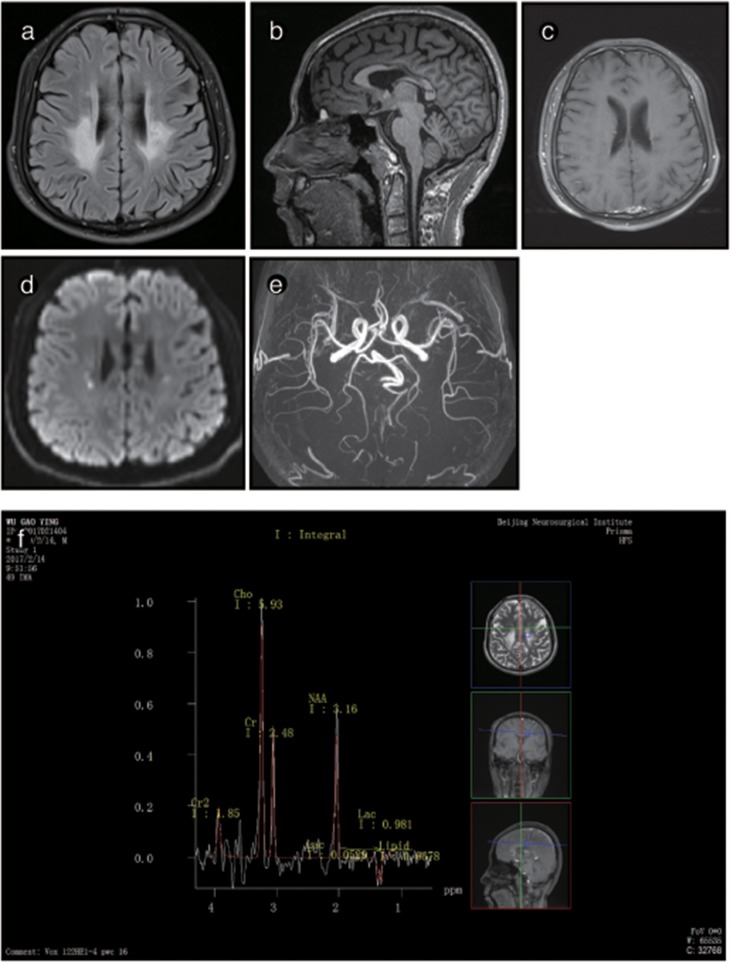
Representative images from patient P2's brain MRI. **(a)** Axial fluid-attenuated inversion recovery (FLAIR) images show a predominant involvement of the frontal and periventricular areas and deep white matter. **(b)** The sagittal T1-weighted image shows multiple hypointense lesions in the corpus callosum. **(c)** Diffusion-weighted imaging (DWI) reveals focal lesions with restricted diffusion in the periventricular white matter. **(d)** A contrast-enhanced MR angiogram shows the normal appearance of the brain vasculature. **(e)** MR angiography (MRA) shows a normal cerebral blood vessel. **(f)** Magnetic resonance spectroscopy (MRS) reveals an inverted lactate peak at 1.33 ppm.

The brain MRI of P3 showed abnormally high signals in the periventricular white matter, the corona radiata, the posterior limb of the internal capsule and the cerebral peduncles. FLAIR images showed leukoaraiosis and atrophy of the corpus callosum body and genu, exhibiting a prominent “sandwich”-like change and suggesting evident cerebellar atrophy. DWI showed paraventricular patchy diffusion restrictions. MRS showed double peaks of lactic acid, and none of the lesions showed any enhancement after the administration of intravenous gadolinium contrast. The brain MRI of P3's mother was normal (Figure 4).

**Figure 5:**
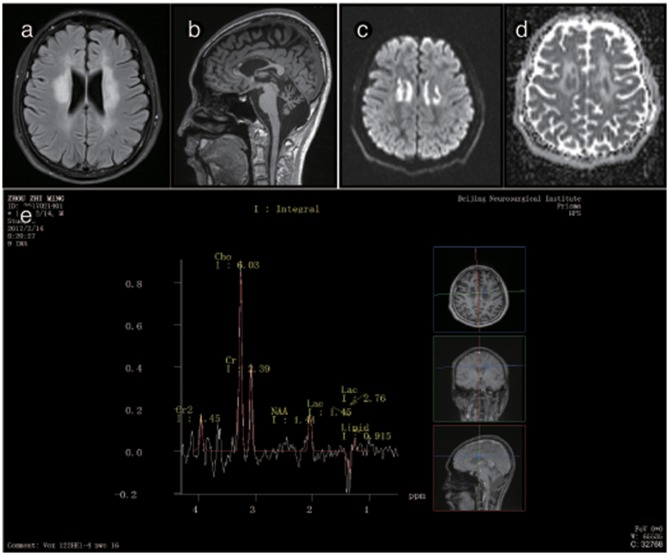
Representative images from patient P3's brain MRI. **(a)** Axial fluid-attenuated inversion recovery (FLAIR) image shows a predominant involvement of the frontal and periventricular areas. **(b)** The sagittal T1-weighted image shows multiple hypointense lesions in the corpus callosum and cerebellar atrophy. **(c)** Diffusion-weighted imaging (DWI) reveals focal lesions with restricted diffusion in the centrum ovale majus. **(d)** The axial average diffusion coefficient (ADC) image shows a periventricular diffusion restricted signal. **(e)** Magnetic resonance spectroscopy (MRS) shows an inverted lactate peak at 1.33 ppm.

**Figure 6:**
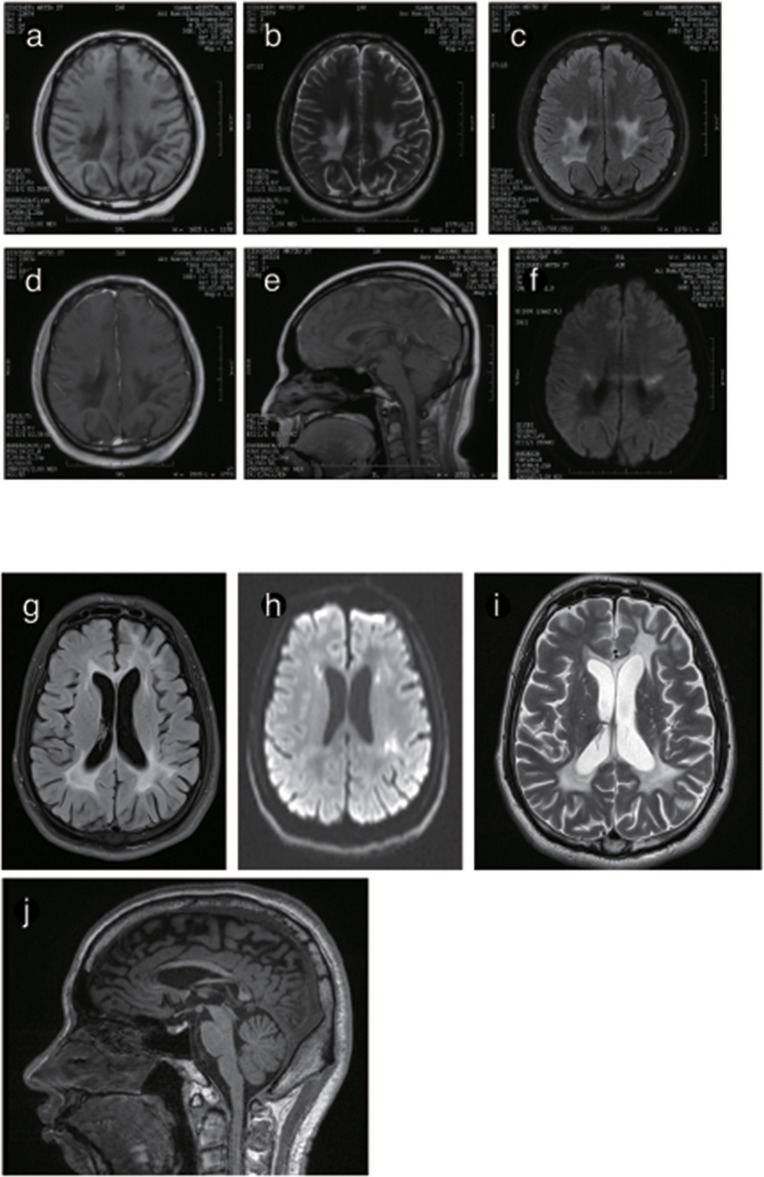
Representative images from patient P4 and P5's brain MRI. **(a)** Axial MRI shows decreased signal intensity on Tl-weighted images, increased signal intensity on T2-weighted images **(b)** and a predominant FLARE signal in periventricular areas with no enhancement **(c)** or ring-like enhancement after contrast injection **(d)**. **(e)** The sagittal T1-weighted image shows multiple hypointense lesions in the postural area of the corpus callosum. **(f)** The ADC image shows a periventricular diffusion restricted signal. **(g)** Axis FLARE image shows asymmetrical patchy lesions and inhomogeneous cerebral white matter abnormalities in the regions around the lateral ventricle **(h)**. The posterior areas of the periventricular white matter show restricted diffusion **(i)**. **(j)** The sagittal T1-weighted image shows that the anterior and posterior segments of the corpus callosum are affected.

The brain MRI of P4 and P5 showed long T1WI and T2WI signals, which were higher but uneven on FLAIR imaging in the posterior horn of the lateral ventricle, the corpus callosum and the posterior limb of the internal capsule. DWI showed restricted diffusion in the posterior horn of the lateral ventricle, the corona radiata, and the posterior limb of the internal capsule. The corpus callosum was obviously shrunken and thinned, and sandwich-like changes could be observed. None of the lesions were enhanced after administration of intravenous gadolinium contrast. The cerebellum showed mild atrophy (Figures 5, 6).

## Discussion

### Main Results

We first reported several novel *AARS2* gene mutations related to late-onset leukodystrophies, except for c.452T>C, p.M151T. All mutations were in the form of the conservation of the genome from zebrafish to humans, which healthy subjects do not carry (Figures 2A,B). These mutations were not described previously, and they could not be found in the ExAC database. All mutations were predicted to be pathogenic by *in silico* analysis using SIFT, PolyPhen-2 and Mutation Taster (Figure 2B). WES showed that 4 of the 5 patients carried *AARS2*-hybrid heterozygous mutations. One patient exhibited an *AARS2* homozygous mutation. The pedigrees of P2 and P4 have clear cases of families with cosegregation and confirmed that the disease was autosomal recessive. The mothers of P2 and P3 carried the same heterozygous gene mutation, namely, c.1871G>A, p.W624X. P2, and P3 were not related and lived 1,000 km from each other.

### Clinical Relevance of Our Results

All four male patients were hospitalized for a detailed investigation. Apart from routine evaluations for acquired causes such as infection, immune disorders, endocrine disorders, poisoning, and tumors, the patients underwent analyses of leukocyte enzymology, very-long-chain fatty acids, amino acids, organic acids, and other common causes of leukodystrophies, such as Krabbe disease, metachromatic leukodystrophy, and adrenoleukodystrophy, among others. The clinical symptoms of these patients included cognitive decline and emotional personality changes (mood swings), dysarthria, nystagmus, ataxia, dystonia, and gait instability. One female patient had ovarian failure, which was consistent with previous reports (17). Interestingly, three male patients had mild abducens nerve palsy, with or without diplopia, and the disappearance of pharyngeal reflexes, which was consistent with the previous literature. In addition, P3 had early-onset developmental delay and decreased exercise capacity. Subsequently, his condition stabilized. After age 20, his condition worsened, and he developed a progressive neurodegenerative syndrome. Recently, other studies reported patients with leukodystrophy as well as optic atrophy, retinopathy, and multiple peripheral demyelinating neuropathies, all of which were considered due to *AARS2* missense mutations (15, 17).

P2 has been diagnosed as having ALSP with typical imaging findings. When we learned that her sister had secondary amenorrhea, she was suspected to have developed vanishing white matter disease (VWM). P3 was diagnosed with spinocerebellar ataxia (SCA) in local hospitals because of apparent ataxia. SCA1, −2, −3, −6, −7, −12, and −17 and dentate-rubro-pallido-luysian atrophy (DRPLA)-related gene mutations were screened by multiple ligation-dependent probe amplification (MLPA). The number of trinucleotide repeats CAG was normal. P4 was treated with corticosteroids for multiple sclerosis in local hospitals without significant improvement.

Brain MRI showed abnormal signals in the periventricular white matter, corpus callosum, and pyramidal tracts in patients, and the lesion area showed a little patchy diffusion limitation, consistent with the pattern of spreading dots in the deep white matter. The corpus callosum showed atrophy in 4 male patients: P2, P3, P4, and P5. The signal was inhomogeneous and resembled a “sandwich”-like change, as reported in previous studies (15, 17). The observation of similar “sandwich”-like changes in the imaging features of this disease requires further analysis. An MRS analysis of P2 and P3 showed an inverted double lactate peak in the lesion area, suggesting a mitochondrial metabolism disorder.

### Comparison of Our Results Within the Context of Existing Literature

*AARS2* consists of 22 exons and encodes a protein of 985 amino acids, a complete structural model of the human mtAlaRS, which includes the arrangement of all three domains: the aminoacylation, editing, and C-terminal domains. Each domain can be further divided into the following subdomains: the aminoacylation domain, containing the subdomains for aminoacylation and tRNA recognition; the editing domain, consisting of the β-barrel and editing core; and the C-terminal or C-Ala domain, consisting of helical and globular subdomains. Several of our mutations were scattered, supporting the random distribution of the disease-causing mutations of this protein (Table 2).

**Table 2:** Adult-onset leukodystrophy-related AARS2 mutations.

**Protein mutations**	**Number of patients**	**Reference**	**Main symptoms**
F50C	1	Dallabona (17)	Gait ataxia, tremor, cognitive decline, psychosis
P60H	1	This study	Consonant dysfunction, slow squint, nystagmus, tremor, walking instability, spasticity, dystonia
A77V	1	Dallabona (17)	Tremor
P131del	2	Dallabona (17)	Tremor, depression, cognitive decline
L193	1	Szpisjak (22)	Psychosis
R199C	7	Lakshmanan (15), Dallabona (17), Szpisjak (22), Lynch (23)	Depression, cognitive decline, psychosis, spastic paraparesis with ataxic signs, anxiety, dysarthria, right upper limb dystonia
P217L	1	Dong (24)	Tremor
Q298delG	2	Lakshmanan (15), Lynch (23)	Cognitive decline, psychosis
Y321	1	Lee (19)	Depression, cognitive deterioration, right hand postural tremor, stooped posture and gait disturbance
T382K	1	Hamatani (18)	Cognitive decline, psychosis
E405K	1	Dallabona (17)	Hemiparesis, ataxia
R521	1	Dallabona (17)	Gait ataxia, tremor, cognitive decline, psychosis
Q537	1	Dallabona (17)	Depression, cognitive decline, psychosis
Q568fs	1	This study	Consonant dysfunction, slow squint, nystagmus, tremor, walking instability, spasticity, dystonia
G570Afs	2	Lakshmanan (15), Lynch (23)	Obsessive behavior, hyperphagia, memory impairment
V730M	4	Dallabona (17)	Depression, cognitive decline, psychosis, spastic paraparesis with ataxic signs
S745Cfs	1	Lynch (23)	Cognitive decline, psychosis
R752fs	1	Lynch (23)	Cognitive decline, psychosis
R756fs	1	Dong (24)	Cognitive decline, psychosis
T871Nfs21	1	Dallabona (17)	Spastic paraparesis with ataxic signs
Q784Sfs	1	Dallabona (17)	Depression, cognitive decline, psychosis
G965R	1	Dallabona (17)	Hemiparesis, ataxia
R963W	2	Dallabona (17) Lee (19)	Depression, cognitive deterioration, right hand postural tremor, stooped posture, and gait disturbance
M151T	4	This study, Lee (19), Sun (25)	Cognitive decline, spastic-ataxic gait, declined memory and calculating ability, bilateral horizontal nystagmus, and hypertonia
M268V	1	This study	Consonant dysfunction, slow squint, nystagmus, tremor, walking instability, spasticity, dystonia
W624X	3	This study, Sun (25)	Spastic-ataxic gait, declined memory and calculating ability, bilateral horizontal nystagmus and hypertonia

### Limitations

In this study, P2 and P3 did not show clear COX-negative fibers, typical RRF and ragged blue fiber (RBF) in the muscle biopsy results, which were similar to those reported in the literature (17). There were no crystalline inclusions under electron microscopy, but a large number of mitochondria accumulated under the sarcolemma in P3. This phenotype suggests the abnormal proliferation of mitochondria. However, we could not observe all the patients' muscle biopsy specimens and electron microscopy images.

Previous studies also showed that progressive white matter dystrophy was associated with decreased activity in mitochondrial complex IV (17). Hence, we performed mitochondrial complex activity examinations on P2 and P3 as well as on healthy controls. Our results indicated that mitochondrial complex IV activity was decreased in P2 and P3 compared with the normal controls. The decrease in mitochondrial complex IV activity in P3 was more prominent, which might be related to a more severe condition. P3 had obvious dysarthria, dysphagia, and walking inability after follow-up for 6 months. However, as previously described, we could not obtain data from all patients.

The genetic diagnosis in this study was conducted through WES instead of whole-genome sequencing (WGS); however, the rate of missed diagnoses with WES is 20%, according to a previous study (26). This is why we have to perform Sanger sequencing to reconfirm these missense variants in these patients. These missense variants in *AARS2* are the most likely cause of these clinical findings. The main reason the patients chose WES instead of WGS was the elevated costs of the WGS procedure. Finally, our findings provide insights into the understanding of *AARS2* genotypic and phenotypic diversity.

## Conclusions and next steps

In short, this study of five patients from four families showed that the *AARS2* mutation causing leukodystrophy with ovarian failure is considered an autosomal recessive inherited disease, supplementing previous reports of scattered cases of deficiencies (17). In addition, the discovery of five pathogenic mutations further confirmed the uncertainty of the *AARS2* gene pathogenesis. It is believed that additional pathogenic sites should be identified.

## Data Availability Statement

All datasets generated for this study are included in the article/supplementary material.

## Ethics Statement

The studies involving human participants were reviewed and approved by Ethics Committee of the Beijing Tiantan Hospital. The patients/participants provided their written informed consent to participate in this study.

## Author Contributions

XW, QW, WS, and ZZ recruited, diagnosed, and assessed patients. XW, WS, QW, HT, BC, XD, SN, SL, and YS worked on the establishment of the separate databases. XW and WS drafted a significant portion of the manuscript or figures. WS and ZZ re-analyzed and interpreted all final data. All authors contributed to the current version of the paper either regarding conception or design, data analysis, or editing, and read and approved the final manuscript.

### Conflict of Interest

The authors declare that the research was conducted in the absence of any commercial or financial relationships that could be construed as a potential conflict of interest.

## Abbreviations

*AARS2*: Alanyl-tRNA synthetase 2
MELAS: Mitochondrial myopathy, encephalopathy, lactic acidosis, stroke-like episodes
MNGIE: Mitochondrial neurogastrointestinal encephalopathy
*ARS2*: Arsenite-resistance protein 2
*DARS2*: Mitochondrial aspartyl-tRNA synthetase
LBSL: Leukodystrophy with brain stem and spinal cord involvement and lactate elevation
OMIM: Online Mendelian Inheritance in Man
OXPHOS: Oxidative-phosphorylation
AARS2-L: Alanyl-transfer RNA synthetase 2 mutation-related leukodystrophy
ALD: Adrenoleukodystrophy
ALSP: Adult-onset leukodystrophy with axonal spheroids and pigmented glia
VWM: Vanishing white matter disease
CADASIL: Cerebral autosomal dominant arteriopathy with subcortical infarcts and leukodystrophy
RRF: Ragged-red fiber
MGT: Modified Gomori trichrome
COX: Cytochrome oxidase
SDH: Succinic dehydrogenase
HE: Hematoxylin-eosin
DWI: Diffusion-weighted imaging
MRA: MR angiography
SWI: Susceptibility weighted imaging
RBF: Ragged blue fiber.
